# Epidemiology of depressive disorders among youth during *Gaokao* to college in China: results from Hunan Normal University mental health survey

**DOI:** 10.1186/s12888-023-04972-w

**Published:** 2023-06-29

**Authors:** Wenhui Yang, Rui Sun, Chong Wang, Jie Chen, Chunguang Zhang, Jie Yu, Haihong Liu

**Affiliations:** 1 Department of Psychology, Center for Cultural Psychology and Behavior Research, Cognition and Human Behavior of Key Laboratory of Hunan Province, Hunan Normal University, 36 Lushan Road, Yuelu District, Changsha, Hunan Province 410081 China; 2 Department of Statistic and Data Science, Cornel University, Ithaca, NY 14853 USA; 3 Student Affairs Department, Center for Education and Mental Health Counsel, Hunan Normal University, 36 Lushan Road, Yuelu District, Changsha, Hunan Province 410081 China; 4 Department of Psychosomatic Medicine, Gerontology of Medical Institute, Academy of Medical Science and People’s Hospital of Guangdong Province, Guangzhou, China

**Keywords:** Depressive Disorders, Adolescent, Young Adult, Age of Onset, Incidence, Prevalence, Risk, Epidemiology, Chinese *Gaokao* (College Entrance exam)

## Abstract

**Background:**

Given the serious consequences of depression and the lack of information about it during the crucially developmental period from the National College Entrance Exam (CEE, i.e., Chinese *gaokao*) to college, this study aimed to estimate the cumulative incidence, prevalence, age of onset, correlates, and service use of depressive disorders (DDs) among youth who passed the CEE and were enrolled at Hunan Normal University in China.

**Methods:**

A two-stage cross-sectional epidemiological survey of DDs was conducted from October to December, 2017 among 6,922 incoming college students (98.5% effective response, N = 6,818, 71.4% female, age range: 16–25 years, mean age = 18.6). Using a stratified sampling method based on the risk of depression, 926 participants (mean age = 18.5, 75.2% female) were selected and subsequently interviewed with the Kiddie–Schedule for Affective Disorders and Schizophrenia–Present and lifetime version (K-SADS-PL).

**Results:**

The sex-adjusted 9-month (i.e., 3 months pre-CEE, 3 months after CEE, and 3 months post-matriculation) incidence of new-onset DDs was 2.3% (standard error [S.E.] 0.3%), and the sex-adjusted 1-month, 6-month and lifetime prevalence were 0.7 (S.E. 0.3%), 1.7 (S.E. 0.2%) and 7.5% (S.E. 1.3%), respectively. The median age of onset was 17 (interquartile range: 16–18) years. Critically, over one-third (36.5%, S.E. 0.6) of depressed youth had their new onset during the 9-month period. The risk factors for depression included having mothers with higher education, experiencing major life events, being female, and experiencing parental divorce or death. The adjusted lifetime treatment rate was 8.7%.

**Conclusion:**

The 9-month incidence of new-onset depression from *gaokao* to college among the youth sample in China is similar to the global annual incidence (3.0%), but the 1-month and lifetime prevalence are significantly lower than the global point (7.2%) and lifetime prevalence (19%). These findings suggest a high proportion of new-onset depression during the CEE to college among the sample youth in China. The risk of depression is associated with familial and stress correlates. Low treatment is a serious concern. Emphasis on early prevention and available treatment for adolescent and young adult depression is a critical need in China.

**Supplementary Information:**

The online version contains supplementary material available at 10.1186/s12888-023-04972-w.

## Introduction

Depressive disorders (DDs) are commonly occurring, serious and recurrent illnesses [1–3]. When depression strikes early in adolescence, it has more serious consequences, often heralding chronic and recurrent problems into adulthood [4–7]. For example, a series of meta-analyses showed that depressed youth have a continued risk for persistence of depressive episodes into adulthood, and a 2-to 3-fold increased odd for adulthood depressive and anxiety disorders [4–7]. Systematic review and meta-analyses also showed that adolescent depression propagates difficulties across the lifespan [8], elevates a significant disease burden [9] and the risk of early death [10, 11]. Longitudinal studies and large-scale meta-analyses on the development of depression showed that middle-to-late adolescence (ages 15–19) has a greater risk for depression onset [12–14]. Therefore, it is worth determining the magnitude, distribution of age of onset, correlates, and receiving treatment of DDs during the high-risk period between late adolescence and early adulthood, especially during the critical transitional period when youth compete for the national college entrance exam (CEE) and admit to college. Then, appropriate prevention and therapeutic programs at the crucial time can be designed to reduce the impact of the disorder before a chronic course is established.

Although prevalence estimates of depression vary with the time period of reference and method of assessment [1], numerous reports have suggested that adolescents and young adults experience high rates of depression [15–18]. A systematic review using data from national surveys on drug use and health in the United States showed that the 12-month prevalence of major depressive episodes was 11.3% in adolescents ages 12–17 and 9.6% in young adults ages 18 to 25 [17]. Data from the WHO Mental Health (WMH) survey showed that the international pooled 12-month prevalence of major depressive disorders (MDD) among college students (ages 18–22) was approximately 4.5–7.7% [15]. In rapidly developing China, a systematic review showed that using clinical diagnostic instruments, the pooled point prevalence of MDD was 1.3% in children and adolescents aged 5–18 [18]. A survey from Jilin University in northeastern China also showed that the lifetime, 12-month, and 1-month prevalence of MDD were 3.9%, 2.4%, and 0.3%, respectively, among college students [16]. In contrast to the numerous epidemiological studies of DDs conducted among either adolescents or early adults, no research has focused on the crucial transition period from pre-CEE [19, 20] to post-matriculation [21] among young people, especially in China.

It is well known that stress [22, 23] and sociocultural factors [24–26] play important roles in the occurrence of adolescent and young adult depression. As 1 in 4 people are aged between 10 and 24, 90% of these young people live in low-income and middle- income countries (LMICs) [27, 28]. In LMICs, young people’s health tends to be more severely affected by cultural, socio-economic and environmental risk factors than in high-income countries, and there are fewer resources to mitigate such risks [28]. Therefore, it is valuable to survey the epidemiology of depression in most of the world’s young people, especially the young people from a large middle-income country of China [29].

Currently, Chinese youth are facing more tremendous socioeconomic transformations than their predecessors. In China, a “cultural trait” effect of deep respect for academic achievements [30] persists in education and economic development [31, 32] and academic tests have a significant function for thousands of years [33]. Up to date, Chinese young people suffer from higher levels of academic stress due to the competitive education system and high parental expectations of academic performance, which are particularly closely associated with high school and college entrance examinations [34–36]. To enter college, Chinese youth need to take the competitive once-a-year national CEE, commonly known as *gaokao*. The *gaokao* is framed as the most important event in Chinese students’ life [32, 37, 38] because the scores of the *gaokao* determine whether one enters college and, if so, what kind of ranked college [37, 39]. To some extent, the college to which one is accepted determines their future job, salary level, and social position or class, thereby affecting their whole life [40–42]. Therefore, students generally prepare for this exam starting at an early age and spend nearly all of their time studying, especially before CEE [43, 44]. Thus, the period from pre-CEE to post-matriculation is the most stressful period for young people because youth during this period need to intensively prepare the CEE [19, 20], apply for college admission [41–43], and then adapt to the independent life after matriculation [21]. Thus, a higher proportion of depression onset might occur in this stage compared to other age periods for Chinese youth.

The high incidence and detrimental consequences of adolescent and young adult depression during this developmental epoch have attracted much concern in China [45, 46]. Over 10 million high school seniors take the CEE to compete for 6–8 million seats through a centralized college admissions system each year [37, 47], and the gross enrollment rate of higher education accounting for the population of this age group from 2017 to 2021 ranged from 45.7 to 57.8% in China [48]. However, to the best of our knowledge, there is a striking lack of the basic information about the distribution of DDs among adolescents and young adults during this critical developmental epoch in China. Thus, it is vital to understand the incidence, prevalence, age of onset, sociopsychological correlates, and service use of DDs among youth who take the CEE and compete for matriculation, so as to precisely design preventive intervention and therapeutic programs before the new onset of adolescent and young adult depression. This could provide crucial information on the optimal timing and targets to policy-makers in China.

In the current study, we conducted a two-stage cross-sectional epidemiological survey among youth who had just passed the CEE and were enrolled at Hunan Normal University (HNU). We aimed to investigate four questions in the sample population to provide basic information for adolescent and young adult depression onset during this crucial developmental period. First, what is the cumulative incidence of DDs during the period from pre-CEE to post-matriculation and the prevalence of DDs among youth who take the CEE and successfully compete for matriculation? Second, what is the distribution of the age of onset, especially the proportion of new-onset DDs from pre-CEE to post-matriculation? Third, what proportion of individuals with DDs have received treatment? Fourth, what sociopsychological factors are associated with the elevated risk of DDs in this critical period?

Given that the most stressful periods during the CEE and matriculation are 3 months before CEE (i.e., the lead up to the exam in the final semester of high school from about March 1st to June 7th [19, 20]), 3 months after CEE (i.e., due to mental conflict regarding the CEE scores and college admission [49]), and 3 months after matriculation (i.e., adapting to the collective life of 1-month military training and sharing a dormitory with 4–12 people [21]), we used a 9-month period (i.e., 3 months pre-CEE, 3 months post-CEE, and 3 months post-matriculation) to capture the most challenging period for depression onset among youth during this transition. To determine the distribution of age of onset among the sample, based on previous studies [13, 50, 51], we divided age of onset into five periods: childhood onset (ages < 12 years), early adolescence onset (ages 12–13 years), mid-adolescence onset (ages 14–15 years), late adolescence onset (ages 16–17 years), and early adulthood onset (ages 18–22 years), to maximize the probability of identifying developmentally and clinically meaningful age-of-onset periods while enabling comparisons with published works.

To identify factors that place youth at risk for experiencing depression, we examined the associations between depressive disorders and a range of important demographic and sociopsychological correlates. In examining family characteristics, studies have shown that family composition and dysfunction are linked to depression, which includes parents’ divorce, single-parent, parental loss [1, 52] and family conflict and abuse [24, 25]). Researchers have also found that mothers’ level of education is associated with offspring depression, although different cultures have different characteristics on this issue [53, 54]. Many studies have documented that, starting in adolescence, the proportion of people who experience this disorder is higher for females than for males [12, 55–57]). Some research, however, has indicated that the sex difference temporarily diminishes in early adulthood [55]. Finally, there is mounting evidence of experiencing major life events during adolescence that make individuals vulnerable to depression [22, 24, 25, 58]. In light of the critical need for the information of adolescent depression onset during the CEE to matriculation in China, the addressed questions would offer the essential information and practical implications for the targeted intervention of adolescent depression.

## Methods

### Setting

This is a cross-sectional two-stage survey of DDs among youth enrolled at Hunan Normal University (HNU). HNU is one of the leading universities and a member of “Project 211” in China. The “Project 211” is the Chinese government’s endeavor aimed at strengthening approximately 100 institutions of higher education (approximately 6%) and key disciplinary areas as a national priority for the 21st century [59]. The “Project 211” universities have been established to meet the national standards of the institutions in terms of overall quality and have adopted a unified admission policy to enroll college students who achieve good CEE scores across China. HNU ranks 89–106 among the ranked 1,211 universities in China [60]. It has 20 colleges and has an enrollment of over 40,000 students for all grades [61], which accounts for 0.24% of Chinese general undergraduates [62].The percentage of the entrants in 2017 (n = 6,922) represented 0.17% of the total entrants of undergraduates (n = 4,107,534 [62]) in China.

### Participants and sampling

The participants in the survey were 6,922 freshmen coming from 31 provincial-level administrative regions across China, which included 22 provinces (excluding Taiwan), 3 autonomous regions (i.e., Inner Mongolia, Xinjiang Uyghur, and Guangxi Zhuang ethics), 2 special administrative regions (Hong Kong and Macao) and 4 municipalities directly under the Central Government (Beijing, Shanghai, Tianjin, and Chongqing). In the province distribution (Supplemental Table 1), 43.3% of the youth came from Hunan Province, 33.2% of the youth came from the other 30 provincial-level administrative regions, and 23.5% were missing this information. In the survey, all freshmen were invited to participate. To increase accessibility to the students, we sent a letter of introduction to the student administrative heads across the 20 colleges for their assistance in the fieldwork. Another letter of introduction was sent to all the students with basic information about the survey and the interviewers’ contact information. Participants were informed that the survey was totally voluntary and they had right to self-determination. All the eligible freshmen (n = 6,922) responded (100% response) and agreed to participate. As 104 participants unfinished the Beck depression inventory-II (BDI-II) (i.e., more than 3 items missing), the effective response rate for screening in the first stage was 98.5% (N = 6,818, 71.4% female, age range: 16–25 years, mean age = 18.6, standard deviation (SD) = 0.82). Among the effective respondents, 42.5% were from cities, 21.7% were from suburban areas, and 35.8% were from rural areas of China. Furthermore, 90.5% were of the Han race, and 9.5% were of other races. For the age distribution and demographic features of the sample, see Table 1. Notably, on the proportion of urban and rural populations and the Han and other races, the sample is consistent with the distributions of the national census [63].

**Fig. 1:**
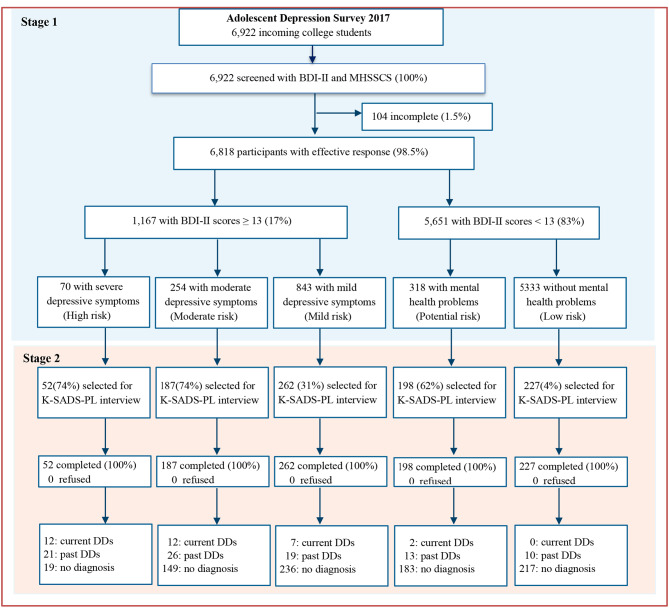
The Flowchart of Procedures of Depressive Disorders Survey among Participants in Hunan Normal University Mental Health Survey BDI-II = Beck Depression Scale-II, MHSSCS = Mental Health Screening Scale for College Students, K-SADS-PL = the Kiddie Schedule for Affective Disorder and Schizophrenia for School-age Children-Present and Lifetime Version, DDs = Depressive Disorders. BDI-II cutoffs: 0–12: minimal; 13–19: mild; 20–28: moderate; and 29–63: severe depressive symptoms. Mental health problems were screened by MHSSCS with cutoffs 2 for any factor scores

For the two-stage survey, we used the census sample in the first screening stage, and then adopted stratified and randomly selected sampling to obtain a representative interview sample in the second stage. Specifically, all the incoming students (n = 6,922) from the 20 colleges sampled and completed the demographic information, Beck Depressive Inventory-II [64, 65] and Mental Health Screening Scale for College Students (MHSSCS) [66] in the first stage. In the second stage, we used the randomized stratification sampling based on the risk of depression. Because depression is a dimensional, not a categorical construct [67–69], depressive symptom severity can provide an optimal way to characterize the quantitative and qualitative aspects of depressive disorders and the overall degree of dysfunction [67–70], so as to provide an effective standard for stratification of sampling. Additionally, as mental health problems are the most common symptoms among youth with subthreshold or remitted depression [71, 72], we combined mental health problems and minimal depressive symptoms to construct the potential and low risk strata of depression. Thus, respondents were classified into five risk strata: (a) the high risk (i.e., with severe depressive symptoms, BDI-II scores 29–63); (b) the moderate risk (i.e., with moderate depressive symptoms, BDI-II scores: 20–28); (c) the mild risk (i.e., with mild depressive symptoms, BDI-II scores: 13–19); (d) the potential risk (i.e., with minimal depressive symptoms, BDI-II scores < 13, and with mental health problem, MHSSCS standard scores ≥ 2); and (e) the low risk (i.e., with minimal depressive symptoms, BDI-II scores < 13, and without mental health problems, MHSSCS standard scores < 2). To maximize the use of relatively limited clinical psychological manpower, we increased proportion of the youth at the potential to high risk among individuals administered the K-SADS in the second stage for sampling design [73, 74]. Specifically, on the basis of depression risk, 52 (74%) youth with severe depressive symptoms were randomly selected from the high risk stratum by simple random methods, 187 (74%) with moderate depression were randomly selected from the moderate risk stratum, 262 (31%) with mild depression were randomly selected from the mild risk stratum, 198 (64%) with minimal depression and mental health problems were randomly selected from the potential risk stratum, and 227 (4%) with minimal depression and without mental health problems were randomly selected from the low risk stratum. Finally, 926 (13.6%) participants were randomly selected, and all agreed to complete the face-to-face clinical interview using the Chinese version of the Kiddie Schedule for Affective Disorder and Schizophrenia for School-age Children-Present and Lifetime (K-SADS-PL) [75–79]. A flow chart describing the stratified sampling and selection and diagnostic interview results is presented in Fig. 1.

**Table 1:** Sociodemographic Features of the Participants in Hunan Normal University Mental Health Survey

Variables	Total sample (N = 6,818)		Structured interview (n = 926)		Comparison ^*a*^	
	*n*	*%*	*n*	*%*	*χ*^*2*^ (*df*)	*p*
Ages					7.6 (5)	0.19
≤ 16	14	(0.2)	2	(0.2)		
17	287	(4.2)	34	(3.7)		
18	3,140	(46.1)	454	(49.0)		
19	2,701	(39.6)	355	(38.3)		
20	564	(8.3)	60	(6.5)		
21–25	112	(1.6)	21	(2.3)		
Sex					5.8 (1)	0.02^*^
Male	1,953	(28.6)	230	(24.8)		
Female	4,865	(71.4)	696	(75.2)		
Residence area					1.9 (2)	0.38
Urban	2,879	(42.5)	410	(44.3)		
Suburban Rural	1,480 2,441	(21.7) (35.8)	185 331	(20.0) (35.7)		
Ethnics					2.1 (1)	0.15
Han	6,169	(90.5)	824	(89.0)		
Others ^b^	649	(9.5)	102	(11.0)		

*Note.*^a^ Compare distribution of each variable between the total sample and the structured interview sample. ^b^ Buyi, Dong, Hasake, Hui, Man, Miao, Mongolian, Sala, Tibetan, Tujia, Uygur, Yao, Yi, Yilao, and Zhuang

^*^Significant sex difference between the two samples *(p* < 0.05)

### Instrument and measures

#### Self-reported demographic-sociopsychological correlates and interviewed growth history

The demographic-sociopsychological correlates included two parts: demographic (by self-report) and individual growth history (by interview). The demographics included (i) date of birth, sex, ethnicity, and residence area (urban, suburban, or rural); (ii) parents’ educational status (primary, junior, senior school, or college); (iii) perceived family economic status (good or moderate, acceptable, or poor or worse); and (iv) family pattern (stable family with two biological parents or remarried parents, parental divorce, or parental loss). The individual growth history included questions on (i) whether or not being left behind at home was experienced during childhood when one or two of the parents were out-of-town due to employment; (ii) whether or not abuse or domestic violence was experienced during childhood; and (iii) whether or not major life events occurred in one’s lifetime (if the answer was positive, life events were demanded to provide). The major life events are defined as events which have a severity of marked threat, lead to large-scale life changes, and directly involve the respondent or the respondent and another person of a significant other [80, 81]. The specific events include (a) “personal” events that happened primarily to the informant, such as sex assault, serious illness or injured, separation by classmates, punishment due to stealing, and (b) “network” events that occurred primarily to, or in interaction with, an individual in the respondent’s social network, such as death, severe illness, jail, permanent disability, fight involving the respondent’s parent, grandparent, or other sibling, major property loss of family (e.g., houses destroyed by floods), or serious trouble getting along with the respondent’s parents.

#### BDI-II

The BDI–II is a 21-item self-report measure of depressive symptoms [64]. Its symptom content reflects the diagnostic criteria for MDD described in DSM-IV. Participants rate the severity of symptoms based on their experiences over the previous 2 weeks on a scale ranging from 0 (low) to 3 (high). Item scores are summed to provide a total score of depression severity. This measure shows sound reliability and validity across a variety of study populations [64, 82] and among Chinese adolescents in this survey [65]. To screen for depressive symptoms, we used BDI-II cutoffs as follows: 0–12, minimal; 13–19, mild; 20–28, moderate; and 29–63, severe depression [64, 65].

#### MHSSCS

The MHSSC is a 96-item self-report measure for screening mental health problems. It scores on a 4-point scale (1 ‘not like me’ to 4 ‘very like me’). The symptom content comprises three dimensions: (1) serious mental health problems (i.e., hallucinations, suicidal ideation or attempts); (2) common mental health problems, including internal problems (i.e., depression, anxiety, social phobia, somatization, etc.) and external problems (i.e., hostility, impulse, obsesses and internet abuse); and (3) developmental problems (i.e., adaptation to college life, relationships and academic pressure). The higher the scores are in a particular dimension, the greater the tendency to exhibit mental health problems in that dimension. We used the MHSSCS standard scores of 2 points to screen mental health problems [66] in participants with minimal depressive symptoms. Correlations between the scores of the screening instruments are presented in Supplemental Table 2.

#### K-SADS-PL for diagnoses of depressive disorders

The K-SADS-PL is a structured clinical diagnostic interview designed to assess the presence and lifetime history of DSM-IV mental disorders in children and adolescents aged 6–18 years [75–79]. There are 82-symptom screening items, and the majority of the items are scored using a 1- to 3-point rating scale. A score of 1 suggests that the symptom is absent, a score of 2 indicates subthreshold levels of symptomatology, and a score of 3 indicates that the symptom is severe and frequent enough to be at or above the threshold. In addition, the K-SADS-PL provides a diagnostic overview of current and lifetime psychopathology, and ratings of global and diagnosis-specific impairment. Diagnoses are scored as definite, probable (≥ 75% of symptom criteria met), or not present.

Based on the DSM–IV-TR Axis I diagnoses, DDs in the survey include major depressive disorder (MDD), dysthymic disorder, and depressive disorder not otherwise specified (NOS), which is mainly minor depressive disorder [83]. To be considered a depressive episode, the symptom cluster had to have lasted at least 1 week for NOS depressive disorder (minor depressive disorder), 2 weeks for major depressive disorder (MDD), 1 year for the dysthymic disorder for youth younger than or equal to age 18 years, and 2 years for youth older than age 18 years, and had to be associated with clinically significant distress or impairment in functioning [83].

Previous work has supported the validity of this instrument when applied in Chinese adolescents with an expanded age of 10–24 years for DD diagnostic screening [84]. The interrater reliability of the K-SADS-PL in the current research was examined among eleven clinical psychological graduates before the survey. The test involved six outpatients with MDD, two outpatients with bipolar disorder, and two outpatients with schizophrenia. The results showed that the kappa value between the interviewers was 0.88 for mental disorders. The validity of the Chinese version for screening DDs in the current study was also assessed among the interviewed youth. The subjects with a positive response to any core symptom of the DDs were treated as screened positive, and the overall sensitivity and specificity of the interview against any diagnostic category of DDs were calculated to be 93–100% and 84–88%, respectively.

We used the modules of affective disorders and psychotic disorders of the K-SADS-PL to evaluate the present, 6-month, and lifetime depressive disorders of DSM–IV-TR Axis I diagnoses and to establish the corresponding 9-month cumulative incidence of the first-onset DDs and 1-month, 6-month, and lifetime prevalence of DDs. The time point (year-month-day) of both onset and ending of the current and past episodes, and the age of the first onset of depressive episode were recorded. During the interview, participants were asked whether they had a time period of persistent dysphoria during the past 6 months, ever before, and what was that now. Symptoms rated in the screen interview were surveyed for *current* and *most severe past* episodes simultaneously. If participants with a history of episodic disorders, a time line was generated to chart lifetime course of the disorder and facilitate scoring of symptoms associated with each episode. Specifically, participants were asked to estimate the age and the date when they first experienced persistent dysphoria coupled with other signs and symptoms of a depressive episode as defined by the DSM-IV, and the timepoint when these symptoms stopped, especially the timepoint before and after the CEE as well as after matriculation. Therefore, the first-onset cases at 3 months before CEE, 3 months after CEE and 3 months after matriculation, and the participants without DDs before these periods could be identified. Meanwhile, information on lifetime treatment, including medicine or any other types, was also collected for depressive disorders. Specifically, individuals were asked if they had used medicine or any other therapy or services for their mental disorders or emotional symptoms during or before the episode when they had depressive symptoms.

### Procedure

The survey was carried out from October 10 to December 13, 2017. In the first stage, all participants were invited to complete the BDI-II in paper-pencil, and then completed the MHSSCS online in computer classrooms in each college during a specified class period. Based on the depressive symptom and mental health problem scores, 926 (13.6%) respondents from 20 colleges were stratified, randomized and invited to participate in the interview in the mental health growth room in each college. During the survey, the safety procedure (i.e., risk management) was conducted in case of adverse events, such as suicidal ideation or suicidal attempt (See Supplemental material 2). Written informed consent for participation was obtained from each participant or the participant’s legal guardian when the participant’s age was less than 18 years before the interview. Supervised by two licensed clinical psychologists, the diagnostic interview was administered by eleven clinical psychological graduates who completed a 40-hour intensive training program for the K-SADS-PL, and two weeks of clinical training, and passed both the diagnostic exams of the DSM-IV and practical tests of the K-SADS-PL. All procedures were performed in accordance with the 1964 Declaration of Helsinki and its later amendments. Ethical approval and the safety procedure were granted by the Research Ethics Committee of Hunan Normal University (protocol code 073).

### Quality control

To ensure a high level of consistency among interviewers, the intensive training program included a detailed course on the use of the K-SADS-PL, the DSM-IV diagnostic classification and criteria, interview skills, role play, mock interviews, patient interviews in hospital, and scoring and interview tests. The training program was closely monitored and supervised by licensed clinical psychologists.

During the survey period, clinical recalibration meetings were held regularly, and all the interviews were audio recorded to estimate interrater reliability. All the interviewers were required to submit the interview records on each working day. The data check on completeness and logicality was completed before the interviewer’s submission. Errors and other flaws were solved both at the scene and at the clinical recalibration meetings.

After the interview, the team leader randomly selected 5% of the records (n = 46) and assigned two blinded clinical psychologists to reassess them with the K-SADS-PL independently. The reliability of diagnoses for current and past mood disorders was excellent (κs = 1.00).

### Statistical analysis

All analyses were conducted using procedures for complex samples. The results of participants who completed the K-SADS-PL assessment were weighted up to the census sample. The initial weight (i.e. sampling weights) for each of the five risk strata (severe, moderate, mild, potential and low risk of depression) were multiplied with the reciprocal of their sampling probabilities for the interview [73, 74]. Poststratification was used on basis of sex (male or female) using data of incoming youth enrolled at Hunan Normal University (Supplemental Table 3). Sex adjustment was used to ensure that the sex distribution of the sample matched the population of college students admitted in the 2017 academic year in China (i.e., 58.0% female) [85]. Cross-tabulation was used to estimate the prevalence, cumulative incidence, the proportional distribution of age of onset across age periods, and during the 9-month period involving the CEE and matriculation. The cumulative incidences of the new-onset DDs were estimated among youth who were at risk. Cumulative incidence and prevalence were reported as a weighted proportion (%) and associated standard error (S.E.). The standard errors were estimated with Taylor-series linearization [86] to adjust for unequal sampling fractions within each risk stratum and possible homogeneity within sampling clusters. The direct standardization method [87] was used for the sex-adjusted incidence and prevalence of DDs, distribution of age of onset, and proportion of new onset across the CEE and matriculation. The associations between DDs and sociopsychological variables were investigated with weighted univariate logistic regression analyses, followed by weighted multivariable logistic regression with variables that showed significant univariate associations. All analyses were conducted with Stata (Stata Corp. 2017, Version 15.0), and tests were performed by a two-sided test (*P* < 0.05). The data are provided in the supplemental materials.

## Results

### The 9-month cumulative incidence and prevalence

Using diagnostic interviews, the 9-month cumulative incidence and prevalence estimates of DDs are presented in Table 2. The sex-adjusted 9-month cumulative incidence of new-onset DDs was 2.3% (Standard error [S.E.] 0.3%). The sex-adjusted lifetime, 6-month, and 1-month prevalence rates were 7.5% (S. E. 1.3%), 1.7% (S. E. 0.2%), and 0.7% (S.E. 0.3%), respectively. The sex-adjusted 9-month incidence of MDD was 1.4% (S.E. 0.3%), and the lifetime, 6-month, and 1-month prevalence rates of MDD were 5.0% (S.E. 1.0%), 1.0% (S.E. 0.2%), and 0.4% (S.E. 0.2%), respectively. The sex-adjusted prevalence of dysthymic disorder was 0.05 (S.E. 0.04%). The sex-adjusted 9-month incidence of DD NOS was 0.9% (S.E. 0.2%), and the lifetime, 6-month, and 1-month prevalence rates were 2.4% (S.E. 0.5%), 0.7% (S.E. 0.1%), and 0.3% (S.E. 0.1%), respectively.

**Table 2:** The 9-month Cumulative Incidence and Prevalence of Depressive Disorders (DDs) among Youth Enrolled at Hunan Normal University (9-month Cumulative Incidence: Weighted N = 6,574, Interviewed Participants without History of DDs, n = 852;Prevalence: Weighted N = 6,818, Interviewed Participants, n = 926)

Diagnosis	9-month cumulative incidence of new onset (%)			Lifetime prevalence (%)			6-month prevalence (%)			1-month prevalence (%)		
	n ^a^	Adjusted^b^ (S.E.%)	Sex-adjusted^c^ (S.E. %)	n ^d^	Adjusted^b^ (S.E.%)	Sex-adjusted^c^ (S.E. %)	n ^d^	Adjusted^b^ (S.E%)	Sex-adjusted (S.E.%)	n ^d^	Adjusted^b^ (S.E.%)	Sex-adjusted^c^ (S.E. %)
**Depressive Disorders**												
Total	48	2.9 (0.3)	2.3 (0.3)	122	6.7 (0.7)	7.5 (1.3)	51	2.5 (0.2)	1.7 (0.2)	33	0.8 (0.5)	0.7 (0.3)
Female	40	3.7^*^ (0.3)		92	5.9 (0.9)		43	3.2^*^ (0.3)		27	1.0 (0.6)	
Male	8	1.1 (0.5)		30	8.7 (2.0)		8	0.8 (0.4)		6	0.5 (0.3)	
**MDD**												
Total	26	1.7 (0.2)	1.4 (0.3)	75	4.1 (0.5)	5.0 (1.0)	32	1.5 (0.2)	1.0 (0.2)	19	0.5 (0.3)	0.4 (0.2)
Female	20	2.0 (0.2)		55	3.2^*^ (0.6)		28	1.8^*^ (0.3)		16	0.6 (0.4)	
Male	6	0.8 (0.4)		20	6.4 (1.6)		4	0.3 (0.2)		3	0.3 (0.2)	
**Dysthymic Disorder**												
Total	-			2	0.04 (0.04)	0.05 (0.04)	2	0.04 (0.04)	0.05 (0.04)	2	0.04 (0.04)	0.05 (0.04)
Female	-			1	0.03 (0.03)		1	0.03 (0.03)		1	0.03 (0.03)	
Male	-			1	0.08 (0.08)		1	0.08 (0.08)		1	0.08 (0.08)	
**Depressive Disorder NOS**												
Total	22	1.3 (0.2)	0.9 (0.2)	45	2.6 (0.3)	2.4 (0.5)	17	1.0 (0.1)	0.7 (0.1)	12	0.3 (0.2)	0.3 (0.1)
Female	20	1.6^*^(0.2)		36	2.7 (0.4)		14	1.3 (0.2)		10	0.4 (0.2)	
Male	2	0.3 (0.3)		9	2.2 (0.8)		3	0.4 (0.3)		2	0.2 (0.1)	

*Note*. ^a^ New onset cases among the interviewed adolescents (n = 852) who did not have DDs at the beginning of the final semester of high school. ^b^ Adjusted with the sampling weights (for female and male estimates) and poststratification weight by sex (for the total estimates) on the data of incoming youth enrolled in Hunan Normal University. ^c^ Sex-adjusted (%) based on Chinese college entrance population (58% female) in the 2017 to 2018 academic year. ^d^ Cases with DDs in the interviewed adolescents (n = 926). MDD = major depressive disorder, NOS = not otherwise specified.

^*^Significant sex difference (*p*s < 0.05)

### Age of onset

Of the 122 youth with current or remitted DDs, all had available age-of-onset data, especially the specific time point of the first onset of DDs during CEE and matriculation. The median age of onset (i.e., the 50th percentile of the age of onset) of DDs was 17 years, and the interquartile range (i.e., the range of ages between the 25th and 75th percentiles) was 16–18 years.

**Fig. 2:**
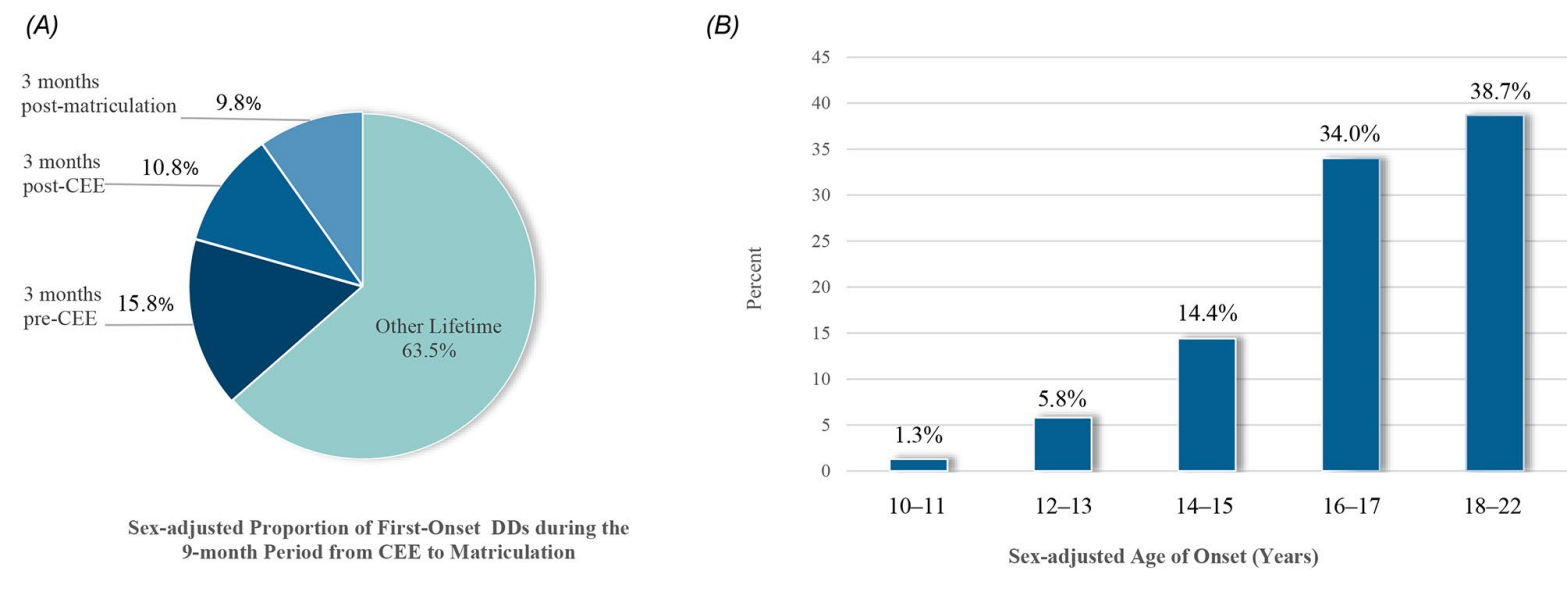
(**A**). Sex-adjusted Proportion of New-onset Depressive Disorders (DDs) During the 9-month Period from the National College Entrance Exam (CEE, i.e., *gaokao*) to Matriculation. (**B**). Sex-adjusted Standardized Distribution of Age of Onset among Youth Enrolled at Hunan Normal University (Percentages do not add up to 100% due to being sex adjusted)

Critically, 39.4% (48/122 cases) of youth with DDs experienced first onset during the 9-month period: 15.6% (19/122 cases) at 3 months pre-CEE, 13.1% (16/122 cases) at 3 months post-CEE, and 10.7% (13/122 cases) at 3 months post-matriculation. The sex-adjusted standardized distribution of the first-onset DDs in the 9-month transition was 36.5% (S.E. 0.6): 15.8% (S.E. 0.4) at 3 months pre-CEE, 10.8% (S.E. 0.4) at 3 months post-CEE, and 9.8% (S.E. 0.4) at 3 months post-matriculation [see Fig. 2 (A)].

Regarding the age of onset, 1.6% (2/122) of youth with DDs had their first onset in childhood (ages 10–11), 6.5% (8/122) in early adolescence (ages 12–13), 14.8% (18/122) in mid-adolescence (ages 14–15), 37.7% (46/122) in late adolescence (ages 16–17), and 39.3% (48/122) in early adulthood (ages 18–22). The sex-adjusted standardized distribution of age of onset was 1.3% (S.E. 0.1) in childhood, 5.8% (S.E. 0.3) in early adolescence, 14.4% (S.E. 0.4) in mid-adolescence, 34.0% (S.E. 0.6) in late adolescence and 38.7% (S.E. 0.6) in early adulthood [see Fig. 2 (B)].

### Sociopsychological correlates of the 9-month incidence, 6-month and lifetime DDs occurrence

In the multivariate analysis (Table 3), the 9-month incidence, 6-month, and lifetime DDs occurrence were all positively correlated with (a) mothers educated for more than 13 years and (b) having experienced major life events. In addition, the occurrence of 6-month DDs and 9-month incidence were both positively correlated with the female sex, whereas the 6-month and lifetime of DDs were both correlated with parental loss. Lifetime of DDs were positively correlated with parental divorce but negatively correlated with fathers educated for more than 7–9 years or 13 years.

**Table 3:** The Crude/Adjusted Odds Ratios (ORs) of Depressive Disorders (DDs) by Sociopsychological Correlates (Weighted N = 6,574 for 9-month New Onset of DDs, Weighted N = 6,818 for Lifetime and 6-month of DDs)

Variables	9-month new onset of DDs			Lifetime of DDs			6-month of DDs		
	*β (Crude/* *Adjusted)*	*Crude OR(95%CI)*	*Adjusted OR(95%CI)*	*β (Crude/* *Adjusted)*	*Crude OR(95%CI)*	*Adjusted OR(95%CI)*	*β (Crude/* *Adjusted)*	*Crude OR(95%CI)*	*Adjusted OR(95%CI)*
Sex									
Male	-	1	1	-	1	-	-	1	1
Female	1.2/1.3	**3.4 (1.2–9.7)**	**3.6 (1.1–11.8)**	-0.4/-	0.7 (0.3–1.3)	-	1.4/1.3	**4.1 (1.5–11.3)**	**3.8 (1.2–12.2)**
Ethnics									
Han	-	1	-	-	1	-	-	1	-
Others	-0.7/-	0.5 (0.1–2.2)	-	-0.7/-	0.5 (0.2–1.6)	-	-0.4/-	0.7 (0.2–2.6)	-
Residence area									
City ^a^	-	1	-	-	1	-	-	1	-
Rural	-0.5/-	0.6 (0.3–1.1)	**-**	-0.5/-	0.6 (0.2–1.4)	-	-0.2/-	0.8 (0.5–1.4)	-
Father’s education years									
1–6	-	1	-	-	1	1	-	1	
7–9	-0.5/-	0.6 (0.3–1.2)	**-**	-0.5/-1.2	**0.6 (0.3–0.9)**	**0.3 (0.1–0.7)**	-0.1/-	0.9 (0.6–1.4)	-
10–12	-0.9/-	0.4 (0.1–2.2)	-	0/-0.4	1.0 (0.6–2)	0.7 (0.3–1.7)	-0.9/-	0.4 (0.1–1.9)	-
13+	-0.2/-	0.8 (0.3–2.2)	**-**	-0.4/-1.2	0.7 (0.4–1.5)	**0.3 (0.1–0.8)**	-0.5/-	0.6 (0.1–2.4)	-
Mother’s education years									
1–6		1	1	-	1	1	-	1	1
7–9	2/2.1	7.3 (0.9–57)	8.1 (0.9–71.9)	1.0/1.5	**2.6 (1.1–5.9)**	4.4 (0.9–21.1)	1.4/1.6	4.0 (0.5–33.6)	4.8 (0.5–45.9)
10–12	0.6/0.7	1.8 (0.5–7.0)	2.0 (0.5–7.8)	-0.2/0.3	0.8 (0.3–2.3)	1.4 (0.5–4.0)	0.5/0.7	1.6 (0.3–7.9)	2.0 (0.4–9.8)
13+	2.3/2.4	**10.1 (2.5–41.5)**	**10.8 (2.5–47.1)**	1.3/2.2	**3.8 (2.3–6.2)**	**9.1 (3.1–27.1)**	1.2/1.5	**3.3 (1.1–9.7)**	**4.4 (1.3–15.6)**
Family economic status									
Good, Moderate Acceptable	-	1	-	-	1	-	-	1	-
Poor or worse	-0.5/-	0.6 (0.1–2.8)	-	-0.9/-	0.4 (0.1–1.4)	-	-0.9/-	0.4 (0.1–1.5)	-
Family patten									
Stable	-	1	**-**	-	1	1	-	1	1
Divorced	0.9/-	2.4 (0.7–8.2)	-	1.1/1.4	**3.0 (1–8.6)**	**4.0 (1.1–14.4)**	1.6/1.6	**4.8 (1–22.7)**	4.9 (0.9–27.4)
Parental death	1.3/-	3.5 (0.8–14.8)	**-**	1.2/1.8	**3.3 (1.1–9.8)**	**6.1 (1.6–23.4)**	2.2/2.3	**9.1 (1.6–51.3)**	**9.5 (1.5–59.3)**
Major life events ^b^									
No	0/0	1	1	-	1	1	-	1	1
Yes	0.7/0.7	**2.0 (1.2–3.3)**	**2.1 (1.2–3.5)**	1.4/1.6	**4.0 (1.9–8.5)**	**4.8 (2.3–10.1)**	0.7/0.9	**2.1 (1.3–3.2)**	**2.5 (1.5–4.2)**
Abused^c^									
No		1	-	-	1	-	-	1	-
Yes	0.8/-	2.3 (0.7–8.2)	-	0.3/-	1.3 (0.5–3.8)	-	0.7/-	2.1 (0.6–7.4)	-
Left behind ^d^									
No		1	-	-	1	-	-	1	-
Yes	-1.2/-	0.3 (0.1–1.5)	-	-1.2/-	0.3 (0.1–1.1)	-	-1.2/-	0.3 (0.1–1.7)	-

*Note.* DDs = depressive disorders. β = standardized logistic regression coefficient. OR = odds ratio. ^a^ City includes urban and suburban. ^b^ Experiencing major life events during childhood or adolescence. ^c^ Experiencing physical abuse or domestic violence during childhood. ^d^ Left behind in hometown during childhood as parents were out-of-town due to employment.

Confidence intervals in bold and underlined indicate statistical significance at *p* < 0.05

### Proportion of treatment

Only 11 (9.0%, 11/122) of youth with DDs had received medication treatment at some point in the past, and greater than 90% (111/122) of the youth with any type of DDs had never received any professional help. The weighted care-seeking behaviors for any DDs was 8.7% (Table 4).

**Table 4:** Unweighted (n = 122) and Weighted (N = 450) Lifetime Rates of Care-seeking Behaviors for Youth with Lifetime Depression

DSM-IV DD	*Total*	Care-seeking behaviors, Unweighted *n* (%)		*Total*	Care-seeking behaviors, Weighted *N*^*a*^ (%)	
		Never sought help	Sought help		Never sought help	Sought help
Any DD	122	111 (91.0)	11 (9.0)	450	411 (91.3)	39 (8.7)
MDD	75	65 (86.7)	10 (13.3)	270	232 (86.0)	38 (14.0)
Dysthymic Disorder	2	2 (100.0)	0 (0.0)	3	3 (100.0)	0 (0.0)
DD NOS	45	44 (97.8)	1 (2.2)	177	176 (99.2)	1 (0.8)

*Note.*^a^ Adjusted for the sampling weights. DSM-IV = diagnostic and statistical manual of mental disorders, the fourth version; DDs = depressive disorders, MDD = major depressive disorder, DD NOS = depressive disorders not otherwise specified

The results of participants who completed the K-SADS-PL assessment were adjusted with sampling weights (i.e., the reciprocal of their sampling probabilities). Poststratification was applied to account for the underrepresented female group in the interview sample (75.2% female, Table 1), and poststratification weight was calculated by utilizing demographic characteristics of sex in census (71.4% female, Table 1). The direct standardization method was used to ensure that the sex distribution of the sample matched the population of college students admitted in the 2017 academic year in China (i.e., 58.0% female). For lifetime rates of care-seeking behaviors, only the sampling weights were applied.

## Discussion

Using a two-stage diagnosed interview focusing on the stressful periods from pre-CEE to post-matriculation, we presented a scarce epidemiological investigation of the cumulative incidence, prevalence, age of onset, psychosocial correlates, and treatment of DDs among youth who took the CEE and enrolled at HUN in China. The findings showed that during the period from *gaokao* to college, the 9-month incidence of DDs was 2.3% (S.E. 0.3%), which is similar to the global annual incidence (3.0% [88]). Critically, over one-third (36.5%) of depressed youth had their first onset during the 9-month period, which indicates a high proportion of new onset depression in the period from the CEE to matriculation among the youth sample. The sex-adjusted 1-month, 6-month and lifetime prevalence were 0.7% (S.E. 0.3%), 1.7% (0.2%) and 7.5% (1.3%), respectively, which are much lower than the global point (7.2% [88] – 8.0% [89]) and lifetime prevalence (19% for MDD [89]). The median age of onset was 17 (interquartile range: 16–18) years. The risk factors for depression included having mothers with higher education, experiencing major life events, being female, and experiencing parental divorce or death. The adjusted lifetime treatment rate was 8.7%. The findings suggest that the transitional period from high school to college is a high-risk time for adolescent and young adult depression onset and some specific sociopsychological risk factors involving depression among youth in China. This information would provide crucial information on the optimal timing and targets to policy-makers for depression prevention in China.

### Prevalence of depressive disorder

The lifetime prevalence rate for DDs in the youth sample was 7.5%, which was comparable to those reported in the recent China Mental Health Survey of community residents aged 18 years or older [90], in which the weighted lifetime prevalence of DDs was 6.8%. In addition, the lifetime prevalence rate of MDD in the sample is similar to that in previous surveys among college students in China [16, 91]. Notably, our data specifically showed that the lifetime prevalence of MDD in male students (6.4%) was significantly higher than that in female students (3.2%). In contrast, the 6-month prevalence and the 9-month cumulative incidence rates in females were significantly higher than those in males. Similar results have been found among college students in China [16, 92]. Further investigation is needed to determine the probable reasons for the higher lifetime prevalence of MDD in male students, but higher 6-month prevalence and 9-month incidence rates in female students in the sample population.

### Age of onset

Regarding the age of onset of DDs, the study showed that the sex-adjusted standardized distribution is 1.3% of the depressed youth onset in childhood, 5.8% in early adolescence, 14.4% in mid-adolescence, 34.0% in late adolescence and 38.7% in early adulthood. Similar patterns have been found in large clinical sample investigations [50] and epidemiological surveys [57], as well as longitudinal studies and the meta-analysis on depression continuities between adolescence and young adulthood [12, 13]. The results may indicate that the period from late adolescence to early adulthood might be high-risk times for first-onset depression across cultures.

### Correlates

Our demographic-sociopsychological correlates of depressive disorders are consistent with those of many previous studies. These results showed that family composition and parental loss during childhood predict later depression [58, 93]. Stress during the past 12 months is the strongest predictive factor for the severity of MDD in college freshmen [94]. Moreover, our results showed that offspring of mothers with more than 13 years of education are associated with an elevated risk for DDs. This finding is in contrast to the results from Western cultures, which indicated that the offspring of mothers with less than a secondary-school level of education were twice as likely to experience a major episode of depression in early adulthood relative to those whose mothers had more education [54]. This might be a cultural characteristic of risk factors for adolescent depression [53]. As noted, parents in Mainland China have been found to be more directive in their parenting and downplay the expression of warmth (i.e., authoritarian), in line with traditional Confucian beliefs in emotional reservedness [95–97]. The probable reason may involve cultural factors impacting the maternal patterns of parenting [98], such as high parental expectations, authoritarianism [53] and control [99], among mothers with higher education in China. The results have a similar pattern as the data of the WMH survey on the association of education years with the risk of depression in China. In Shenzhen, the least educated individuals had the lowest risk of major depressive episodes (MDEs), whereas in high-income countries, the least educated had the highest risk of MDEs [100].

Interestingly, our findings simultaneously showed that lifetime DDs were negatively correlated with fathers educated for more than 7–9 years or 13 years. This might reflect cultural factors impact on patterns of parenting of mothers and fathers in China, and their association with DDs. As a series of cross-cultural comparisons between Chinese and Western parents [101–104] showed that Chinese mothers and fathers were more authoritarian (i.e., physical coercion, verbal hostility, and nonreasoning oriented regulation) than their Western counterparts. Somewhat surprisingly, researchers found that Chinese fathers were rated as less authoritarian than Chinese mothers [103]. In addition, on perceived parenting and risk for major depression in Chinese women[53], researchers also found that the pathogenic effect of maternal authoritarianism was stronger than that of paternal authoritarianism for risks of MDD. Furthermore, for protectiveness (i.e., an overprotective and controlling parental style), the researchers surprisingly found that paternal protectiveness was negatively associated with risk for MDD while maternal protectiveness was positively associated with risk for MDD[53]. High parental protectiveness is generally pathogenic in Western countries but protective in China, especially when received from the father [53]. This further supported cultural factors impact on patterns of parenting of mothers and fathers, and their risk for DDs in China. Replication is needed.

### Treatment rate

Unfortunately, we show that the treatment rate of depressive disorders was considerably low in youth in the sample, which suggests that the low service utilization for depression is a greater problem in young people in China. Again, these findings are consistent with the previous data from four provinces [74], the national epidemiological survey from China [90], and the WMH survey in developing countries [15, 105]. Possible reasons for this may include the stigma associated with depression, cultural response bias partly due to stoicism—a relatively high tolerance for or denial of emotional suffering of depression [106], and the scarcity of access to service due to the fact that available services are concentrated in urban-based specialty psychiatric hospitals, and most suburban and rural areas have little or no access to mental health services [74, 107, 108].

### Prevention of adolescent and young adult depression

Transitioning from high school to college is an important developmental milestone that holds the potential for personal growth and behavioral change [109]. The evidence showed that negative life experiences in this transition period are uniquely associated with depressive symptom trajectories even after adjusting the effects of adolescent characteristics [110]. Specifically, negative and stressful life experiences maintain or rapidly elevate depressive symptoms during this developmental period [111]. As the period from high school to college involves a number of significant life-transition experiences, reducing negative life experiences and increasing positive life experiences is vital. However, the evidence showed that the vast majority (86.1%) of young people during 4–10 weeks before taking the CEE experienced moderate to extreme stress and 43.6% had depressive symptoms in Taiwan [19]. Numerous studies have shown that 16.7–35.4% of Chinese freshmen experience moderate to severe depressive symptoms in the first year [21, 91, 112, 113], especially at 3 months after admission [21]. Our findings also showed 16.6% (S.E. 0.5%) sex-adjusted prevalence of self-reported depressive symptoms, 11.5% (S.E. 0.4%) self-reported suicidal ideation and 5.8% (S.E. 1.3%) suicidal ideation by interview among the youth sample (Supplemental Tables 4 and Supplemental Fig. 1). Given the far-reaching consequences of adolescent and young adult depression, timely and effective intervention need be taken before the new onset.

Given that this study focused on young people who successfully passed the CEE and enrolled at HUN, the rates of new-onset depression might be higher on those who took the CEE but failed to enroll at the university. There are two aspects of this evidence. On the one hand, a series of studies demonstrated that returnees who failed the CEE had serious mental health problems compared to their peers in China [114–117]. On the other hand, the WMH surveys, including a Chinese adolescent sample, demonstrated that college students had a significantly lower MDD prevalence than nonstudents in the same age range from 18 to 22 [15].

Given the early age of onset (median 17 years) and a high proportion of new-onset depression during the period from the CEE to matriculation, depression might have serious adverse effects on this critical developmental transition. For example, previous results from a WMH survey showed that mental disorders with pre-matriculation onset were more important than those with post-matriculation onset in predicting subsequent college attrition [15]. As depression is associated with a significantly elevated disease burden [9], post-dysfunctional harms [118, 119] and the risk of early death [10, 11], screening youth in high-risk times (ages14–22), from middle-to-late adolescence to early adulthood (Fig. 2B), might prevent the disorders and decrease harm early. Additionally, the subsequent offer of specific interventions such as evidence-based cognitive behavior treatment (CBT) [120, 121] and negative attentional bias modification training [122] to high-risk youth or those who have depressive symptoms (indicated prevention) might result in a more developmental approach to the prevention of depressive disorders before its peak onset. For those with moderate-to-severe depressive disorders, the strong evidence supported that psychotherapy (e.g., CBT) combining with pharmacotherapy (e.g., fluoxetine) seems to be the best choice [123, 124]. For the likely cost-effectiveness of expanded depression prevention and treatment from a societal perspective, an allocation of mental health resources may focus on innovative, low-threshold, inexpensive, and scalable interventions, such as computerized cognitive training [125], to prevent and treat adolescent depression [125] in large, scalable self-help procedures. This may ultimately help reduce this disorder’s large burden and alleviate its post-dysfunctional harms in youth.

Admittedly, the scarcity of access to and availability of some treatments (notably CBT) for depression in non-specialist contexts is a major concern in low-income and middle-income countries [126, 127]. Currently, the Action Plan to Develop Specialized Services for the Prevention and Treatment of Depressive Disorders [128] in China is a significant policy in the progress of mental health services. However, to implement the Plan, several major challenges need be addressed, such as lack of resources, lack of united action, and insufficient awareness of depression [129, 130]. Thus, prevention and treatment for depression, especially the improved access of services is a long-term process cross low-income and middle-income countries [131], including China [129, 130].

### Limitations

Although our study is the first investigation that uses the K-SADS-LP, a standardized diagnostic tool, to assess the incidence, first-onset age, distribution of depression, specific sociopsychological risk factors, and service of use for depression among Chinese youth in the pivotal developmental period, several limitations need be noted.

First, as the survey was conducted in one of the “211” universities, caution should be taken when generalizing the data, even if a previous study showed that no difference in the distribution of depressive disorders among youth across the key and ordinary universities in China [91]. Specifically, although it is an accurate estimate of the new-onset DD rates with high response (98.5%) and interview rates (100%) among the census sample of youth passing CEE to HUN, it might only represent depression distribution, its correlates, and treatment for the sample youth. Second, the incidence and age of onset were estimated through diagnostic interviews of participants’ reports, so recall bias is inevitable [132]. Even a salient marker of the CEE was used to improve the accuracy of recalling symptoms and disorder onset in critical periods. Third, using a retrospective approach, the distribution of age of onset among youth with depression might lead to definitive results [133]. In other words, there might be a potential bias on the age of onset of depression due to the restricted age of the youth sample. Fourth, although the results were adjusted for the sex ratio according to the national data, the sample population consisted of a large proportion of females. Fifth, the cross-sectional nature of the surveys makes it impossible to determine the temporal direction of associations between demographic-sociopsychological variables and depressive disorders.

Despite these limitations, the results present some valuable information on the depression distribution and onset in the pivotal epochs between late adolescence and early adulthood. It also reveals the specific high-risk period for the onset of depression and specific social-cultural risk factors for depression among youth in China. Meanwhile, the research reconfirms findings from previous epidemiological studies.

## Conclusion

Overall, a high proportion of new-onset depression during the period from pre-CEE to post-matriculation, familial and stress correlates, and lack of treatment were found in the sample youth in China. The evidence from this survey posts serious challenges related to the need of early identifying and intervening in depression among youth in China. The findings provide key information for policy-makers and healthcare professionals to explore and address factors that affect adolescent and young adult depression in this pivotal developmental period.

## Electronic supplementary material

Below is the link to the electronic supplementary material.

Supplementary Material 1: Supplementary Material 1 (Excel file): Data of epidemiology of depression from gaokao to college in Chinese youth.

Supplementary Material 2: Supplementary Materia 2 (Word file): **The safety procedure in case of adverse events** and supplemental tables: **Table S1** Province distribution of the participants, **Table S2** Correlations matrix of screening Instruments, **Table S3** The 9-month cumulative incidence and prevalence of depressive disorders (DDs) with sampling weight and poststratification weight, **Table S4** the prevalence of depressive symptoms and suicide ideation, and **Figure S1** the flowchart of procedures of suicidal ideation survey.

## Data Availability

All data generated or analysed during this study are included in this published article [and its supplementary information files].
