# Supplementary material for: Epidemiology of depressive disorders among youth during Gaokao to college in China: results from Hunan Normal University mental health survey
Source: BMC Psychiatry. 2023 Jun 29;23:481. doi: 10.1186/s12888-023-04972-w (PMC10308668; doi:10.1186/s12888-023-04972-w)
Supplement: Supplementary file 2 — Supplementary Material 2: Supplementary Materia 2 (Word file): The safety procedure in case of adverse events and supplemental tables: Table S1 Province distribution of the participants, Table S2 Correlations matrix of screening Instruments, Table S3 The 9-month cumulative incidence and prevalence of depressive disorders (DDs) with sampling weight and poststratification weight, Table S4 the prevalence of depressive symptoms and suicide ideation, and Figure S1 the flowchart of procedures of suicidal ideation survey. [file 12888_2023_4972_MOESM2_ESM.docx]

Supplemental materials

**The Safety Procedure in Case of Adverse Events**

If suicidal ideation or suicidal attempt of a participant was identified by item 9 (suicidal ideation) of the BDI-II or item 9b (suicidal ideation) of major depression episode of the K-SADS-PL (Supplemental Table 4), a risk assessment would be further conducted by staff member of the HNU mental health center, who would follow safety protocols of the university.

To make service available, all participants were provided the service phone number of the HNU mental health counsel center (0731-88872843) and Contacting Us information before interview. A guide of coping with depression and a list of mental health resources about emergency services were also provided. In addition, the depressed youth were encouraged to seek for help, e.g., contact a relative, call the mental health center for counseling, or visit the psychiatric service in hospitals directly.

# Supplemental Table 1

*The Province Distribution of the Participants in Hunan Normal University Mental Health Survey.*

| Province | n (%) |
| --- | --- |
| 1. Anhui | 143 (2.1) |
| 1. Fujian | 73 (1.1) |
| 1. Gansu | 56 (0.8) |
| 1. Guangdong | 92 (1.4) |
| 1. Guizhou | 41 (0.6) |
| 1. Hainan | 33 (0.5) |
| 1. Hebei | 138 (2.0) |
| 1. Heilongjiang | 72 (1.1) |
| 1. Henan | 133 (2.0) |
| 1. Hubei | 134 (2.0) |
| 1. Hunan | 2,954 (43.3) |
| 1. Jiangsu | 132 (1.9) |
| 1. Jiangxi | 136 (2.0) |
| 1. Jilin | 53 (0.8) |
| 1. Liaoning | 63 (0.9) |
| 1. Qinghai | 25 (0.4) |
| 1. Shandong | 134 (2.0) |
| 1. Shanxi | 118 (1.7) |
| 1. Shanxi | 73 (1.1) |
| 1. Sichuan | 96 (1.4) |
| 1. Yunnan | 38 (0.6) |
| 1. Zhejiang | 128 (1.9) |
| 1. Beijing | 32 (0.5) |
| 1. Chongqing | 81 (1.2) |
| 1. Shanghai | 24 (0.4) |
| 1. Tianjin | 73 (1.1) |
| 1. Guangxi Zhuang ethics | 63 (0.9) |
| 1. Inner Mongolia | 18 (0.3) |
| 1. Xinjiang Uyghur | 48 (0.7) |
| 1. Hong Kong | 9 (0.1) |
| 1. Macao | 5 (0.07) |
| Unknown | 1,600 (23.5) |

# Supplemental Table 2

*Correlations matrix (the Pearson’s r) of Screening Instruments during the Two-Stage Survey (n=926).*

| Variables | BDI-II | MHSSCS | K-SADS-PL | |
| --- | --- | --- | --- | --- |
|  |  |  | Current MDE | Past MDE |
| BDI-II | 1 |  |  |  |
| MHSSCS | 0.22^***^ | 1 |  |  |
| K-SADS-PL: Current MDE | 0.28^***^ | 0.15^***^ | 1 |  |
| Past MDE | 0.27^***^ | 0.12^***^ | 0.57^***^ | 1 |
| *Mean (SD)* | 14.19 (8.63) | 93.29 (46.53) | 9.84 (2.35) | 11.4 0(3.91) |

*Note.* BDI-II = Beck Depressive Inventory-II, MHSSCS = Mental Health Screening Scale for College Students, K-SADS-PL = Kiddie Schedule for Affective Disorder and Schizophrenia for School-age Children-lifetime, MDE = Major Depression Episode, Current MDE = total scores of the 9 items for depressive symptoms assessed by current MDE module of K-SADS-PL (score range: 9-27), Past MDE = total scores of the 9 items for depressive symptoms assessed by past MDE module of K-SADS-PL (score range: 9-27).

*** *P* < 0.001

# Supplemental Table 3

*The 9-month Cumulative Incidence and Prevalence of Depressive Disorders (DDs)with Sampling Weight and Poststratification Weight by Sex on the data of Incoming Youth Enrolled at Hunan Normal University ( 9-month Cumulative Incidence: Interviewed Participants without History of DDs, n=852, Weighted N=6,574；Prevalence: Interviewed Participants, n=926, Weighted N=6,818).*

| Diagnosis | 9-month cumulative  incidence of first onset (%) | | | | Lifetime prevalence (%) | | | 6-month prevalence (%) | | | | | 1-month prevalence (%) | | | |
| --- | --- | --- | --- | --- | --- | --- | --- | --- | --- | --- | --- | --- | --- | --- | --- | --- |
|  | n ^a^ | Sampling  weight ^b^  (S.E.%) | | Poststratification  weight ^c^  (S.E.%) | n ^d^ | Sampling  weight ^b^  (S.E.%) | Poststratification weight^c^ ^c^ (S.E.%) | n ^d^ | | Sampling  weight ^b^  (S.E.%) | | Poststratification  weight^c^ (S.E%) | n ^d^ | Sampling  weight ^b^  (S.E.%) | Poststratification  weight^c^ (S.E.%) | |
| **Depressive Disorders** | | | |  |  |  |  |  | | | | |  |  | |  |
| Total | 48 | 3.1 (0.3) | | 2.9 (0.3) | 122 | 6.6 (0.7) | 6.7 (0.7) | 51 | | 2.6 (0.2) | | 2.5 (0.2) | 33 | 0.8 (0.5) | | 0.8 (0.5) |
| Female | 40 | 3.7^*^ (0.3) | |  | 92 | 5.9 (0.9) |  | 43 | | 3.2^*^ (0.3) | |  | 27 | 1.0 (0.6) | |  |
| Male | 8 | 1.1 (0.5) | |  | 30 | 8.7 (2.0) |  | 8 | | 0.8 (0.4) | |  | 6 | 0.5 (0.3) | |  |
| MDD |  |  | |  |  |  |  |  |  | |  | |  |  | |  |
| Total | 26 | 1.8 (0.2) | | 1.7 (0.2) | 75 | 4.0 (0.6) | 4.1 (0.5) | 32 | | 1.5 (0.2) | | 1.5 (0.2) | 19 | 0.5 (0.3) | | 0.5 (0.3) |
| Female | 20 | 2.0 (0.2) | |  | 55 | 3.2^*^ (0.6) |  | 28 | | 1.8^*^ (0.3) | |  | 16 | 0.6 (0.4) | |  |
| Male | 6 | 0.8 (0.4) | |  | 20 | 6.4 (1.6) |  | 4 | | 0.3 (0.2) | |  | 3 | 0.3 (0.2) | |  |
| Dysthymic Disorder |  |  | |  |  |  |  |  | |  | |  |  |  | |  |
| Total | - |  | |  | 2 | 0.04 (0.04) | 0.04 (0.04) | 2 | | 0.04 (0.04) | | 0.04 (0.04) | 2 | 0.04 (0.04) | | 0.04 (0.04) |
| Female | - |  | |  | 1 | 0.03 (0.03) |  | 1 | | 0.03 (0.03) | |  | 1 | 0.03 (0.03) | |  |
| Male | - |  | |  | 1 | 0.08 (0.08) |  | 1 | | 0.08 (0.08) | |  | 1 | 0.08 (0.08) | |  |
| Depressive Disorder NOS | |  |  |  |  |  |  |  | |  | |  |  |  | |  |
| Total | 22 | 1.3 (0.2) | | 1.3 (0.2) | 45 | 2.6 (0.3) | 2.6 (0.3) | 17 | | 1.1(0.2) | | 1.0(0.1) | 12 | 0.3 (0.2) | | 0.3 (0.2) |
| Female | 20 | 1.6^*^(0.2) | |  | 36 | 2.7 (0.4) |  | 14 | | 1.3(0.2) | |  | 10 | 0.4 (0.2) | |  |
| Male | 2 | 0.3 (0.3) | |  | 9 | 2.2 (0.8) |  | 3 | | 0.4(0.3) | |  | 2 | 0.2 (0.1) | |  |

*Note*. ^a^ New onset cases among the interviewed youth (n = 852) who did not have DDs at the beginning of the final semester of high school. ^b^ Adjusted estimates with the product of sampling weights. ^c^ Adjusted estimates with sampling weights and poststratification weight by sex on the data of incoming youth enrolled in HNU (71.4% female). ^d^ Cases with DDs in the interviewed adolescents (n = 926). MDD = major depressive disorder, NOS = not otherwise specified. ^*^Significant sex difference (*p*s < .05).

*Supplemental Table 4*

*The Prevalence of Depressive Symptoms and Suicide Ideation among Youth Enrolled at Hunan Normal University (Screened Participants: N = 6,818; Interviewed Participants, n=926, Weighted N=6,818)*

| Diagnosis | Prevalence (screened) | | | Prevalence (interviewed) | | |
| --- | --- | --- | --- | --- | --- | --- |
|  | n ^a^ | % | Sex-adjusted^b^ (S.E.%) | n ^c^ | Sampling  weight ^d^  (S.E.%) | Sex-adjusted^b^ (S.E.%) |
| Depressive symptoms ^a^ |  |  |  |  |  |  |
| Total | 1167 | 17.1 | 16.6 (0.5) | - |  |  |
| Mild | 843 | 12.4 | 12.1 (0.4) |  |  |  |
| Moderate | 254 | 3.7 | 3.5 (0.2) | - |  |  |
| Severe | 70 | 1.0 | 1.0 (0.1) | - |  |  |
| Suicidal ideation ^a^ |  |  |  |  |  |  |
| Total | 815 | 12.0 | 11.5 (0.4) | 83 | 5.6 (1.4) | 5.8 (1.3) |
| Female | 632 | 13.0 |  | 64 | 5.5 (1.7) |  |
| Male | 183 | 9.4 |  | 19 | 6.0 (1.6) |  |

*Note*. ^a^ depressive symptoms: scores of Beck Depression Inventory-II (BDI-II) ≥ 13, suicidal ideation: item 9 (suicidal ideation) scores of BDI-II ≥1. ^b^ Sex-adjusted (%) based on Chinese college entrance population (58% female) in the 2017 to 2018 academic year. ^c^ item 9b (suicidal ideation) scores of major depression episode from the Kiddie Schedule for Affective Disorder and Schizophrenia for School-age Children-Present and Lifetime Version (K-SADS-PL) ≥ 2 (n = 83). ^d^ Adjusted estimates with the product of sampling weights using poststratification based on the risk of suicidal ideation [high risk: scores of item 2 (hopeless) or item 9 (suicidal ideation) of BDI-II ≥ 1, low risk: scores of both item 2 and item 9 of BDI-II = 0], for sampling weights see supplemental Figure1.

Supplemental Figure 1

**Stage 1**

**Stage 2**

429 completed (100%)

0 refused

497 completed (100%)

0 refused

104 incomplete (1.5%)

**Adolescent Depression Survey 2017**

6,922 incoming college students

6,818 complete screening (effective response rate 98.5%)

1,502 with item 2 (hopeless) or item 9 (suicidal ideation) of BDI-II ≥ 1 (22%)

(High risk of suicidal ideation)

5,316 with both item 2 and item 9 of BDI-II = 0 (78%)

(Low risk of suicidal ideation)

497 (33%) selected for

K-SADS-PL interview

429 (8%) selected for

K-SADS-PL interview

69: suicide ideation

428: no diagnosis

14: suicide ideation

416: no diagnosis

6,922 screened with BDI-II (100%)

BDI-II = Beck Depression Scale-II, K-SADS-PL = the Kiddie Schedule for Affective Disorder and Schizophrenia for School-age Children-Present and Lifetime Version.

*Supplemental Figure 1. The Flowchart of Procedures of Suicidal Ideation Survey among Participants in Hunan Normal University Mental Health Survey*
